# Adaptation of *Saccharomyces cerevisiae* Cells to High Ethanol Concentration and Changes in Fatty Acid Composition of Membrane and Cell Size

**DOI:** 10.1371/journal.pone.0002623

**Published:** 2008-07-09

**Authors:** Thai Nho Dinh, Keisuke Nagahisa, Takashi Hirasawa, Chikara Furusawa, Hiroshi Shimizu

**Affiliations:** 1 Department of Biotechnology, Graduate School of Engineering, Osaka University, Suita, Osaka, Japan; 2 Department of Bioinformatic Engineering, Graduate School of Information Science and Technology, Osaka University, Suita, Osaka, Japan; Baylor College of Medicine, United States of America

## Abstract

**Background:**

Microorganisms can adapt to perturbations of the surrounding environment to grow. To analyze the adaptation process of the yeast *Saccharomyces cerevisiae* to a high ethanol concentration, repetitive cultivation was performed with a stepwise increase in the ethanol concentration in the culture medium.

**Methodology/Principal Findings:**

First, a laboratory strain of *S. cerevisiae* was cultivated in medium containing a low ethanol concentration, followed by repetitive cultivations. Then, the strain repeatedly cultivated in the low ethanol concentration was transferred to medium containing a high ethanol concentration and cultivated repeatedly in the same high-ethanol-concentration medium. When subjected to a stepwise increase in ethanol concentration with the repetitive cultivations, the yeast cells adapted to the high ethanol concentration; the specific growth rate of the adapted yeast strain did not decrease during repetitive cultivation in the medium containing the same ethanol concentration, while that of the non-adapted strain decreased during repetitive cultivation. A comparison of the fatty acid composition of the cell membrane showed that the contents in oleic acid (C_18:1_) in ethanol-adapted and non-adapted strains were similar, but the content of palmitic acid (C_16:0_) in the ethanol-adapted strains was lower than that in the non-adapted strain in media containing ethanol. Moreover, microscopic observation showed that the mother cells of the adapted yeast were significantly larger than those of the non-adapted strain.

**Conclusions:**

Our results suggest that activity of cell growth defined by specific growth rate of the yeast cells adapted to stepwise increase in ethanol concentration did not decrease during repetitive cultivation in high-ethanol-concentration medium. Moreover, fatty acid content of cell membrane and the size of ethanol-adapted yeast cells were changed during adaptation process. Those might be the typical phenotypes of yeast cells adapted to high ethanol concentration. In addition, the difference in sizes of the mother cell between the non-adapted and ethanol strains suggests that the cell size, cell cycle and adaptation to ethanol are thought to be closely correlated.

## Introduction

Cells of microorganisms can adapt to environmental perturbations, such as changes in osmotic pressure and temperature, and depletion of nutrients. The yeast *Saccharomyces cerevisiae* has been used for the production of useful chemical compounds as well as alcoholic beverages. In the industrial production of useful target products using yeast cells, cells have faced a variety of the environmental changes, such as an increase in osmotic pressure, the accumulation of ethanol and carbon dioxide and a decrease in the amount of nutrients [1]. Of these environmental changes, the accumulation of ethanol during cultivation causes stress to yeast cells, leading to a decrease in cell growth and the production of target products. Thus, understanding the adaptation process of yeast under high ethanol concentration is important and this might lead to construction of the yeast strains that can grow well in high ethanol concentration and are highly desired in the field of production of useful compounds using cells.

Some researchers have analyzed adaptation phenomena of yeast cells to high ethanol concentration. Lloyd et al. found that yeast previously grown in the presence of 5% ethanol could grow in the medium containing 10% ethanol, whereas yeast inoculated directly into medium containing 10% ethanol failed to grow [2]. Ismail and Ali reported that no increase in the tolerance of yeast to a high ethanol concentration was observed after ten successive transfers to an environment containing a high ethanol concentration [3]. Therefore, it is expected that exposing yeast cells to a stepwise increase in the level of ethanol stress will be effective for obtaining the ethanol-tolerant yeast strains.

In this study, we performed a repetitive cultivation with a stepwise increase in ethanol concentration to observe the adaptation process of *S. cerevisiae* to a high ethanol concentration. For this purpose, a laboratory strain of *S. cerevisiae*, FY834, was cultured in medium containing a low ethanol concentration, followed by repetitive cultivation. After that, the cells adapted to the low ethanol concentration were inoculated into medium containing a higher ethanol concentration, followed by repetitive cultivation with a stepwise increase in the ethanol concentration. By exposing to a stepwise increase in ethanol concentration in the culture medium, cell growth activity defined by specific growth rate (SGR) of the yeast cells did not decreased during repetitive cultivation in high-ethanol-concentration medium. This indicates that the yeast cells successfully adapted to high ethanol concentration. In addition, analyses of fatty acid composition of the cell membrane and cell morphology of the ethanol-adapted strains were also performed. The results of these analyses suggest that change in fatty acid composition and increased cell size might be the typical phenotypes of ethanol-adapted yeast cells.

## Results and Discussion

### Effect of ethanol addition on cell growth of *S. cerevisiae*

**Figure 1:**
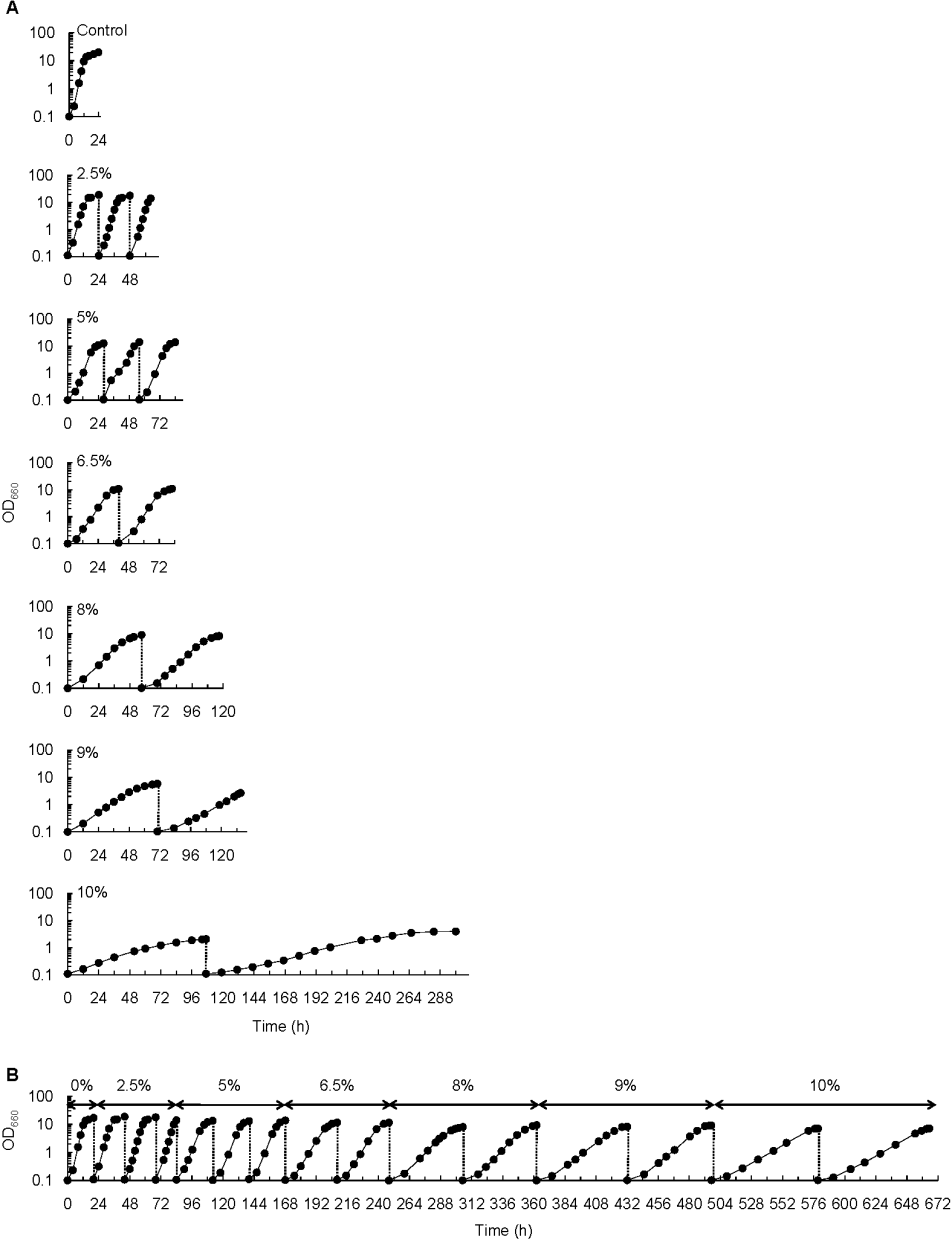
Growth characteristics of FY834 under high ethanol concentrations. (A) Growth characteristics of FY834 in repetitive cultivations at high ethanol concentrations. FY834 was inoculated into medium containing 2.5, 5, 6.5, 8, 9 or 10% ethanol, respectively, and then the growth of each culture was monitored, measuring OD_660_. In each culture, the culture broth was transferred to fresh medium containing the same ethanol concentration and then the growth was observed. The timing of the transfer to fresh medium is shown by the dotted lines. (B) Growth characteristics during subjection of FY834 to stepwise increase in ethanol concentration with repetitive cultivations. The method used for subjecting the yeast cells to a stepwise increase in ethanol concentration is described in Materials and methods. The timing of the transfer of culture to fresh medium is shown by the dotted lines. In the experiment shown in this figure, we obtained an ethanol-adapted strain series A2.

To evaluate the effect of ethanol on yeast cell growth, a laboratory strain of *S. cerevisiae* FY834 was directly inoculated into YPD medium containing 2.5, 5, 6.5, 8, 9, or 10% (v/v) ethanol, respectively, and then the cells were incubated at 30°C (Figure 1A). After that, the culture was transferred to medium with the same ethanol concentration. The cell density for inoculation was set to about 10^6^ cells/ml, because the inoculum size affects the length of the lag time of yeast cell growth as reported by Walker-Caorioglio et al. [4].

The SGRs during the repetitive cultivation in which the cells were directly inoculated into medium with a high ethanol concentration are summarized in Table 1. In the YPD medium, the SGR of FY834 was 0.45±0.04 h^−1^ and decreased as a result of increase in ethanol concentration. The SGR further decreased in the second cultivation, compared with that in the first cultivation in medium with the same ethanol concentration. This phenomenon was clearly observed when ethanol concentration was more than 6.5%, that is, the difference in SGR between the first and second cultivations became large.

**Table 1:** Effect of ethanol addition on growth of *S. cerevisiae* strain FY834

Cultivation	Specific growth rate at each ethanol concentration (h^−1^)						
	0%	2.5%	5%	6.5%	8%	9%	10%
First	0.45±0.04	0.39±0.020	0.27±0.015	0.19±0.026	0.13±0.017	0.09±0.011	0.04±0.004
Second	-	0.38±0.005	0.26±0.006	0.15±0.020	0.11±0.010	0.06±0.015	0.03±0.006
Third	-	0.38±0.010	0.26±0.005	-	-	-	-

Average of SGR±standard deviation of three independent experiments in each culture condition is shown.

### Adaptation of *S. cerevisiae* to high ethanol concentration during stepwise increase in ethanol concentration in culture medium

Adaptation to environmental changes is achieved by the mechanism that a cell adjusts its intracellular physiological conditions to the surrounding environment to grow. Ismail and Ali reported that adaptation does not occur when the environmental changes are extreme [3]. However, yeast cells are expected to be able to grow well even in the severe environments, when intracellular physiological conditions can be appropriate for growth in such environments through the stepwise adaptations to the environmental changes. Therefore, we analyzed the adaptation process of yeast to a high ethanol concentration through a repetitive cultivation of *S. cerevisiae* with a stepwise increase in the ethanol concentration.

The FY834 strain was subjected to a stepwise increase in the ethanol concentration of the medium from 2.5 to 10% via 5, 6.5, 8 and 9% through the repetitive cultivation, and the SGR in each cultivation process was measured. We independently obtained four series of ethanol-adapted strain, named A1, A2, A3 and A4. As an example, the growth of yeast strain series A2 during stepwise adaptation process is shown in Figure 1B. Similar growth properties were observed in the series A1, A3 and A4 to that in series A2 (data not shown). As shown in Table 2, the SGR of ethanol-adapted strain series decreased in the first culture with an increase in the ethanol concentration of the medium. Unlike the case of Table 1, the SGR of the second cultivation became almost the same as that of the first cultivation. This result suggests that the yeast cells once subjected to certain ethanol concentration are able to adapt to the same condition. Even though the ethanol-adapted strains showed a decrease in SGR with increase in ethanol concentration in the medium, a relatively high SGR was maintained in the yeast cells experiencing the cultivation with stepwise increase in ethanol concentration. Here, the strain showing the phenotype in which the SGR does not decrease through repetitive cultivation with a stepwise increase in ethanol concentration is called the ethanol-adapted strain.

**Table 2:** Specific growth rates of ethanol-adapted yeast strain series during stepwise increase in ethanol concentration with repetitive cultivation

Cultivation	Specific growth rate at each ethanol concentration (h^−1^)						
	0%	2.5%	5%	6.5%	8%	9%	10%
First	0.45±0.032	0.38±0.009	0.26±0.006	0.18±0.014	0.12±0.028	0.08±0.012	0.06±0.001
Second	-	0.37±0.005	0.26±0.003	0.17±0.015	0.12±0.020	0.09±0.006	0.06±0.002
Third	-	0.39±0.004	0.26±0.007	-	-	-	-

Average of SGR±standard deviation of four ethanol-adapted strain series in each culture condition is shown.

We next compared the SGR in the second cultivation of the ethanol-adapted yeast strain series (Table 2) with that in the second cultivation of the non-adapted yeast strain (Table 1). In case of medium containing more than 6.5% ethanol, the SGR of ethanol-adapted strain became larger than that in non-adapted strain. However, in the case of lower than 6.5%, the difference in SGRs between ethanol-adapted and non-adapted strains in the presence of same ethanol concentration was not significant.

### Comparison of fatty acid compositions of cell membrane between non-adapted and ethanol-adapted strains

Some researchers have shown that the cell membrane is one of the primary targets of ethanol: Alterations in the lipid composition of the cell membrane affect the ethanol tolerance of yeast and unsaturated fatty acids result in an increase in membrane fluidity and an enhancement of ethanol tolerance [5]–[10] Therefore, we analyzed the fatty acid compositions of the cell membrane and compared them among ethanol-adapted yeast strains in media containing ethanol.

**Figure 2:**
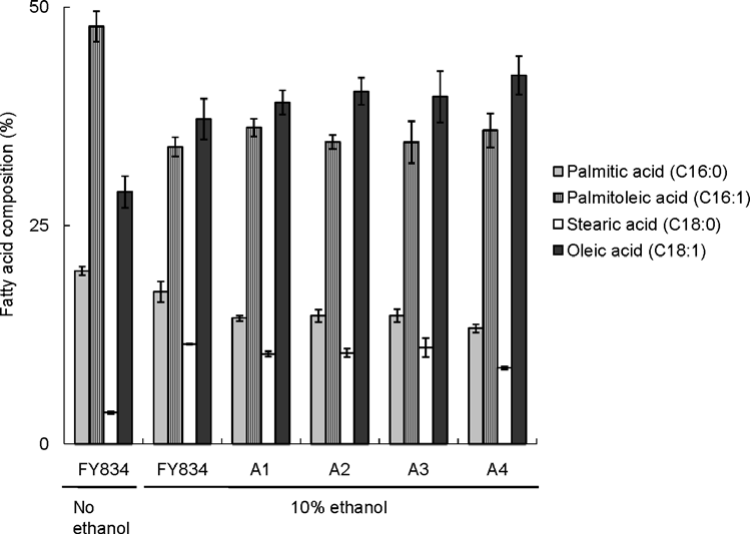
Comparison of fatty acid compositions of yeast cell membrane among four adapted strains. Ethanol-adapted strain series were cultivated in YPD medium in the presence of 10% ethanol and the fatty acid composition of each strain was determined. Fatty acid compositions of FY834 strain cultured in the presence and absence of ethanol were also measured. Data represented the average values of three independent experiments with standard deviations.

The content of oleic acid (C_18:1_) in FY834 strain grown in the presence of 10% ethanol was higher than that of the FY834 grown in the medium without ethanol addition (Figure 2). However, the content of oleic acid in the cell membrane of the ethanol-adapted strains was similar to that of the non-adapted yeast grown in the presence of 10% ethanol, while the fraction of C_16:0_ fatty acids was lower in adapted yeast strains than in the non-adapted strain in the presence of 10% ethanol (Figure 2). These results suggest that the fatty acid composition in cytoplasmic membrane was changed by exposing yeast cells to high ethanol concentration, but the increased fraction of C_18:1_ fatty acid was not observed in the ethanol-adapted strain compared with the non-adapted strain. However, decreased fraction of C_16:0_ fatty acid in ethanol-adapted strains was found in the presence of 10% ethanol, suggesting that the content of other fatty acid(s) that could not be measured in this study might be changed during adaptation process to high ethanol concentration.

### Comparison of cell size between non-adapted and ethanol-adapted strains

**Figure 3:**
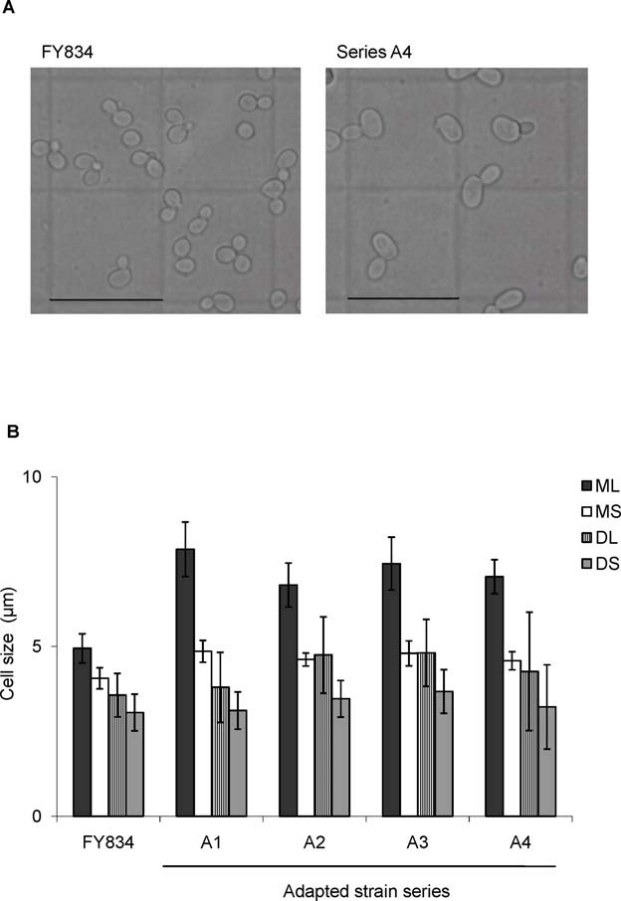
Cell morphology of control and ethanol-adapted yeast strain series. The yeast cells obtained through the repetitive cultivation with a stepwise increase in the ethanol concentration were cultivated in the YPD medium and the cell morphology was observed. (A) Microscopic observation of cell morphologies of ethanol-adapted strain series A4 and non-adapted yeast strain (FY834). The bars in each photograph represent 25 µm. (B) Comparison of cell size between non-adapted strain and adapted strain series at mid-log phase. ML, the longest diameter of mother cell; MS, the shortest diameter of mother cell; DL, the longest diameter of daughter cell; DS, the shortest diameter of daughter cell. Data represent the average size of counted 50 cells with standard deviations.

Since Kubota et al. showed that the size of the cells cultured in the presence of ethanol was enlarged [11], we observed the morphology of ethanol-adapted and non-adapted strains cultured in the presence of 10% ethanol. The yeast cells exposed to 10% ethanol through a stepwise increase in ethanol concentration in each culture series were inoculated into medium without the addition of ethanol. After reaching the mid-log phase, the morphologies of the cells were microscopically observed. The cells at mid-log phase were used for microscopic observation because at this phase yeast cells have adapted to new condition of medium. It is thought that during batch cultivation the cells achieve the “steady state” at the mid-log phase. The cells of the ethanol-adapted strains became larger than those of in the non-adapted strain (Figure 3A).

To quantitatively evaluate the change in cell morphology, the longest and shortest diameters of the cells of ethanol-adapted and non-adapted strains were compared (Figure 3B). The shortest diameters of the mother and daughter cells in the non-adapted and ethanol-adapted strains were similar. In contrast, the longest diameter of cells was different between the ethanol-adapted and the non-adapted strains, namely, a difference in the longest diameters of the mother cells between the non-adapted and ethanol-adapted strains was clearly observed. The longest diameter of the mother cells of the ethanol-adapted strains was larger than that of the non-adapted strain. These phenomena were consistent with the results shown by Kubota et al. [11].

In the budding yeast *S. cerevisiae*, it is well known that the cells must achieve the critical cell size [12]. The cell division of *S. cerevisiae* is asymmetric, i.e. one small daughter cell is budded from one mother cell during one cell division event. In principle, the size of the mother cell is always achieved the critical cell size, indicating that the size of mother cell is independent on the cell cycle. In contrast, the size of daughter cell is dependent on the cell cycle, because the daughter cell has to enlarge to achieve the critical cell size for initiating cell division during cell cycle progression. Enlargement of the mother cell size of the ethanol-adapted strains shown in this study suggests that the critical cell size of the ethanol-adapted strains might be changed to adapt to high ethanol concentration. The change in cell size might be one of the typical phenotype of ethanol-adapted yeast cells.

### Conclusion

In this study, the stepwise adaptation process of *S. cerevisiae* was investigated by carrying out exposure to a stepwise increase in ethanol concentration with repetitive cultivations. When subjecting yeast cells to a stepwise increase in the ethanol concentration of the culture medium, the SGR of the ethanol-adapted strain did not change through repetitive cultivation under high ethanol concentration, suggesting that we can successfully obtain a strain, whose SGR is not changed through repetitive cultivation at high ethanol concentrations. Observations of cell morphology and measurement of fatty acid composition of the cell membrane in adapted and non-adapted yeast strains revealed that yeast cells change the intracellular physiological state, such as a change in the composition of fatty acid(s) that could not be measured in this study and the increase in the size of yeast, to adapt to ethanol stress. These might be the typical phenotypes of ethanol-adapted yeast.

From the observation results of the difference in sizes of the mother cell between the non-adapted and ethanol strains, the cell size, cell cycle and adaptation to ethanol are thought to be closely correlated. Enlargement of the mother cell size of the ethanol-adapted strains shown in this study suggests that the critical cell size of the ethanol-adapted strains might be changed to adapt to high ethanol concentration. Moreover, the relationship between the stress response and the cell cycle of yeast has been also suggested [for review, see 13]. Thus, it might be important to analyze the relationship between the cell cycle progression and adaptation to high ethanol concentration. Furthermore, the relationship between the difference in growth rates between non-adapted and ethanol-adapted strains under high ethanol concentration condition and cell cycle progression should be also investigated.

In addition, another factors related to the adaptation to high ethanol concentration should be taken into account, such as fatty acids in the cell membrane that could not be observed in this study, lipids content in the cell membrane and ergosterol that is one of the component of cell surface and responsible for ethanol stress tolerance in *S. cerevisiae* [5]–[10], [14], [15]. Analyses of these factors as well as cell cycle progression in ethanol-adapted strains will help us to understand the molecular mechanism of adaptation to high ethanol concentration in *S. cerevisiae*.

## Materials and Methods

### Strain, medium and cultivation conditions

*Saccharomyces cerevisiae* FY834 (*MAT*α *his3*Δ*200 ura3-52 leu2*Δ*1 lys2*Δ*202 trp1*Δ*63*) [16] obtained from the yeast genetic resource center (Osaka University, Japan), was used in this study. Yeast cells were precultured in 5 ml of YPD medium (1% Bacto yeast extract, 2% Bacto peptone, 2% glucose) in a test tube at 30°C for 24 h. Then, for the main culture, the precultured yeast was cultivated aerobically at 30°C in a Sakaguchi flask containing 100 ml of YPD medium. The cell density for inoculation was set to about 10^6^ cells/ml, because the inoculum size affects the length of the lag time of yeast cell growth as reported by Walker-Caorioglio et al. [4].

### Repetitive cultivation of *S. cerevisiae* with stepwise increase in ethanol concentration

Repetitive cultivation with a stepwise increase in ethanol concentration was performed as follows. The cultivation of FY834 strain was carried out in YPD medium containing ethanol and then the culture was transferred to fresh medium containing the same ethanol concentration. After that, the culture was transferred to medium containing a higher ethanol concentration, followed by repetitive cultivations. The initial ethanol concentration was set at 2.5% (v/v) and it was changed to 5, 6.5, 8, 9 and 10% step by step. In cases of cultivations with 2.5 and 5% ethanol in the medium, the transfer of the broth at the end of the previous culture to medium containing the same ethanol concentration was performed twice. In cases of the cultivations containing 6.5, 8, 9 and 10% ethanol, the transfer of the culture was performed once. We performed four series of such repetitive cultivations from 2.5% of ethanol concentration to 10%, and the obtained culture series were called A1, A2, A3, and A4, respectively.

The adaptation of the yeast was evaluated by measuring the optical density of the culture at 660 nm (OD_660_) and calculating SGR of all the strains examined using OD_660_ data at mid-exponential phase. The SGR is defined as increasing rate of cell concentration (OD_660_) divided by cell concentration, by calculating the slope of the logarithmic plot of OD_660_ value as described by Blanch and Clark [17].

### Measurement of fatty acid composition of cell membrane

Yeast cells exposed to the stepwise adaptation process were cultivated in YPD medium containing 10% ethanol and harvested at the mid-log phase by centrifugation. After the cells were washed twice with sterilized water, they were lyophilized for one day using the lyophilizer (VD-500F; Taitec, Japan). The extraction of fatty acids and their methyl esterification were carried out as described by Hama et al [18]. Analysis was performed using a gas chromatograph (GC-3000; Hitachi, Japan) with a column, TC-FFAP (30 m×0.25 mm i.d., 0.5 µm phase thickness; GL Science, Japan). The analytical conditions used for gas chromatography were as follows. The initial temperature of the column was 120°C. The column temperature was increased to 240°C at 10°C/min and then maintained for 10 min [19]. Injector and detector temperatures were 250 and 280°C, respectively. Nitrogen gas was used as a carrier. Fatty acid methyl ester mixtures (Sigma-Aldrich Inc, catalog number 18918) were used as lipid standards. The peaks corresponding to each fatty acid methyl ester in the obtained samples were identified comparing their retention times with those of the lipid standards.

### Measurement of the size of yeast cells

Yeast cells exposed to the stepwise adaptation process were cultivated in YPD medium and the cells at the mid-log phase (OD_660_ = about 1) was observed using phase contrast microscope (BX60, Olympus). Microphotographs of yeast cells were taken by a digital camera (Camedia C-5060 Wide Zoom, Olympus). For each strain, long and short diameters of mother and daughter cells from 50 cells were measured. For evaluation of the difference in cell sizes between the ethanol-adapted and non-adapted strains, average and standard deviation of long and short diameters of mother and daughter cells, respectively, from 50 cells were calculated.
